# Sandfly Blood-Feeding Habits and Competence in Transmitting Ntepes Virus, a Recently Discovered Member of the Genus *Phlebovirus*

**DOI:** 10.1155/2022/4231978

**Published:** 2022-10-20

**Authors:** Epaphrus Yuko, Rosemary Sang, Eunice A. Owino, Johnstone Ingonga, Damaris Matoke-Muhia, Iman B. Hassaballa, Sandra Junglen, David P. Tchouassi

**Affiliations:** ^1^International Centre of Insect Physiology and Ecology, P.O. Box 30772-00100, Nairobi, Kenya; ^2^University of Nairobi, P.O. Box 30197-30100, Nairobi, Kenya; ^3^Kenya Medical Research Institute, P.O. Box 54840-00200, Nairobi, Kenya; ^4^Institute of Virology, Charité Universitätsmedizin Berlin, Corporate Member of Free University Berlin, Humboldt-University Berlin, and Berlin Institute of Health, Chariteplatz 1, 10117 Berlin, Germany

## Abstract

Phleboviruses transmitted by sandflies are among emerging public health threats. A novel *Phlebovirus* named Ntepes virus (NTPV) was recently described and found to infect humans from a wide geographic area in Kenya. However, the entomologic risk factors of this virus such as the potential vectors and the transmission cycles remain poorly defined. This study assessed the ability of the colonized sandfly *Phlebotomus duboscqi* to transmit NTPV and determined the bloodmeal host sources of field-collected sandflies from the area where NTPV was found in Baringo County, Kenya. Five-day old laboratory-reared *P. duboscqi* were orally challenged with an infectious dose of NTPV (≈10^6.0^ pfu/ml) and incubated for up to 15 days postinfection. Individual sandflies were dissected into abdomens, legs, and salivary glands and screened for the virus infection by cell culture. Of the 205 virus-exposed sandflies, 19.5% developed non-disseminated infections in the midgut, with no evidence of virus dissemination or transmission in legs and salivary glands, respectively. The midgut infection rates decreased with increasing extrinsic incubation period (Spearman's correlation, *ρ* = −0.71). Blood-fed specimens analyzed by polymerase chain reaction (PCR) and sequencing of a region of the mitochondrial 12S rRNA, revealed almost exclusive feeding on humans (98%) represented by the sandflies *Sergentomyia schwetzi*, *S. clydei*, *S. antennata, S. squamipleuris, S. africana,* and *Phlebotomus martini.* One specimen of *S. clydei* had fed on cattle (2%). These findings suggest *P. duboscqi* is an incompetent laboratory vector of NTPV. The high human-feeding rate by diverse sandfly species increases the likelihood of human exposure to pathogens associated with these sandflies. Assessment of the susceptibility of *Sergentomyia* species to NTPV is recommended given their high human-feeding tendency.

## 1. Introduction

Sandfly-associated *Phlebovirus* infection in humans cause self-limiting acute febrile illness (AFI) collectively known as sandfly fever and infections of the nervous system [1–3]. In sub-Saharan Africa, due to the paucity of active surveillance, poor disease reporting systems, and lack of appropriate diagnosis, sandfly fever can be misdiagnosed as other AFI-like diseases like malaria and influenza [2, 4]. In Kenya, recent studies have detected novel sandfly-associated phleboviruses, some closely related to viruses associated with febrile illness such as sandfly fever Naples and sandfly fever Sicilian virus serogroups [5, 6]. In the absence of active surveillance, the public health impact of these infections could be underestimated.

Ntepes virus (NTPV) has been isolated from sandflies in 2014 and 2016 in Baringo County, Kenya [5, 6] and established the novel *Ntepes phlebovirus* species within the genus *Phlebovirus,* family *Phenuiviridae*. Serological analysis showed evidence of human infection based on the detection of neutralizing antibodies against NTPV in human serum samples. The virus appears to have a wide distribution in Kenya based on human seroprevalence data in areas beyond its isolation focus. Ntepes virus has repeatedly been detected in sandflies from the ecology where it was first isolated [5] indicating that NTPV could be endemic in Kenya. Ntepes virus was also shown to potentially exhibit a wide vertebrate host range based on cell culture infection experiments [6]. Nonetheless, our understanding of the transmission ecology remains poor including potential reservoirs of the virus in nature. Sandflies of the genus *Sergentomyia* have been implicated in the circulation of NTPV based on its detection in naturally infected sandfly pools [5, 6]. However, the identification of vectors involved in transmission requires data beyond the sole detection of a virus in a vector.

Sandflies in the genus *Phlebotomus* have largely been associated with the transmission of sandfly fever viruses in the Old World and Mediterranean region [1, 5–9] including *Phlebotomus papatasi, P. perfiliwei, andP. pernicious* [8]. Experimental infection studies have demonstrated sandflies from diverse species including *P. papatasi, P. duboscqi, P. sergenti,* and *Lutzomyia longipalpis* as competent vectors of the mosquito-borne *Phlebovirus* Rift Valley fever virus [10–12]. Few other *Sergentomyia* species can harbor phleboviruses in nature [1, 5] or have been shown to act as competent vectors in laboratory experiments [12].

*Phlebotomus papatasi* of the subgenus *Phlebotomus rondani* is the major incriminated vector of sandfly fever viruses in sub-Saharan Africa [13]. *Phlebotomus duboscqi* is the only representative of this subgenus in the NTPV focus in Baringo, Kenya [14], and a primary vector of zoonotic cutaneous leishmaniasis [15, 16]. A close relationship between leishmaniasis and sandfly-borne phleboviruses has been proposed, following cooccurrences in the same ecological areas [17, 18] and the likelihood that both diseases share common vectors [18]. The involvement of *P. duboscqi* in the transmission of NTPV could have ramifications for possible epidemiological relationships between NTPV and leishmaniasis in the NTPV foci as has been reported for leishmaniasis and phleboviruses transmitted by *Phlebotomus* vectors [19, 20].

The transmission of arboviruses in a given ecology is governed by the intricate interactions among the pathogen, competent arthropod vector, and a susceptible vertebrate host in a conducive environment [21, 22]. Vector competence assesses the physiological permissiveness of a species to a virus infection in the midgut, its replication, and successive dissemination to the salivary glands of the vector [23]. Assessment of the vector competence of suspected vector species to emerging phleboviruses serves as a fundamental step in understanding their transmission ecology and providing relevant information on disease risk and preparedness for epidemics as well as vector control. On the other hand, the identification of bloodmeal sources of hematophagous arthropods provides information on their host-feeding preferences which may lead to the identification of vertebrate reservoirs for specific pathogens, their route of transmission [24], and guide the design of control strategies.

In this study, we sought to understand the entomological risk factors of the transmission of NTPV by investigating the capacity of laboratory colonized *P. duboscqi* to transmit NTPV after oral infection through artificial membrane feeding. We also analyzed the blood-host feeding patterns of feral sandfly species in the NTPV foci area to evaluate the potential risk of human exposure to viruses they might transmit.

## 2. Materials and Methods

### 2.1. Sandfly Samples

The adult female *P. duboscqi* used for the vector competence experiment were obtained from a preestablished colony (in 2010) at the vector biology insectary of the Kenya Medical Research Institute (KEMRI), Nairobi, Kenya. The sandflies were reared on apple slices for sugar sources *ad libitum* and mouse blood provided thrice a week as anesthetized mice in polycarbonate cages at 26°C and 80% relative humidity with a photoperiod of 12 h:12 h. Archived blood-fed field-collected sandfly specimens preserved at -80°C at the International Centre of Insect Physiology and Ecology (icipe) Duduville Campus, Nairobi, were used for bloodmeal source analysis. The samples were surveyed in December 2018 and January 2020 in Rabai, Baringo County, Kenya; the area where NTPV was isolated [6]. The sandflies were trapped using CDC light traps from selected habitats indoors (households) and outdoors (around termite mounds and animal sheds). Details of the trapping design are described by Hassaballa et al. [25].

### 2.2. Ntepes Virus Stock and Amplification

Cryopreserved stock of NTPV (at icipe) isolated from a pool of sandflies in Marigat, Baringo, Kenya in 2014 [6] was used for vector competence experiments. The NTPV stock was passaged in Vero cells. Working stocks were cultured in Vero cells and quantified before use. Briefly, the virus was grown on a monolayer of Vero cells in a T-25 cell culture flask on minimum essential medium (MEM) (Sigma-Aldrich, St. Louis, MO) supplemented with 10% heat-inactivated fetal bovine serum (FBS), 2% L-glutamate, 2% antibiotic/antimycotic solution composed of 1000 parts of penicillin, 25 micrograms of amphotericin B, and 10 mg streptomycin per ml and incubated at 37°C in 5% CO_2_ until the cells attained an 80%-90% confluence on the culture flask. Two hundred microliters (200 *μ*l) of the stock NTPV were inoculated onto the confluent monolayer of Vero cells and then incubated for 1 h with periodic gentle agitation for virus adsorption onto the cells. The infected Vero cells were then maintained for up to 14 days in MEM augmented with 2% heat-inactivated FBS, 2% antibiotic/antimycotic solution, and 2% L-glutamate and observed for cytopathic effects (CPE) daily. Once 80% of the monolayer appeared to show CPE, the flask was frozen overnight at -80°C, thereafter, thawed on wet ice and clarified by centrifugation at 3000 rpm for 10 min. The resulting supernatant was harvested and aliquoted in 1.5 ml cryovials and stored at -80°C for further use.

### 2.3. Virus Quantification

The amplified virus was quantified by plaque assay by inoculating 100 *μ*l of serial 10-fold dilutions of the harvested virus onto each well of a confluent monolayer of Vero cells in the 12-well culture plate that had been seeded with a growth medium 24 h before virus inoculation. The inoculated virus was incubated for 1 h with periodic agitation for virus adsorption. The NTPV-inoculated cells were maintained on 2% methylcellulose overlay mixed with 2× MEM (GIBCO Invitrogen Corporation, Carlsbad, California) and incubated at 37°C with 5% CO_2_ for 10 days, then fixed for 1 h with 10% formalin and stained for 2 h with 0.5% crystal violet. The plaques were counted and the virus titer was quantified using the formula described by Brady et al. [26];

(1) $$\begin{array}{c} \text{PFU}=\frac{\text{Number of plaques}}{\text{Dilution factor x Volume of diluted virus}}. \end{array}$$

### 2.4. Experimental Infection of Sandflies with NTPV

A total of 528 five-day-old female *P. duboscqi* in three replicates were offered an infectious bloodmeal containing 10^4.3^ to 10^6.6^ pfu/ml of NTPV (Table 1). The viremic bloodmeal was prepared by mixing 5 ml of the harvested NTPV with 5 ml defibrinated sheep blood (1 : 1). Shaven mouse skin served as a membrane to cover the wells of feeder maintained on a hemotek membrane feeder (Discovery Workshops, Accrington, UK). The NTPV-viremic blood was applied into the well of the feeding system (2 ml/well) maintained at 37°C. Prestarved sandflies (24 h) were allowed to feed for 1 h in darkness. Before and after exposure of the sandflies, an aliquot of the viremic bloodmeal (1 ml) were stored immediately at -80°C for further virus quantification by cell culture.

The blood-fed sandflies were left to set in the infection cages for at least 24 h to allow the fragile peritrophic membrane covering the bloodmeal to harden. Fully engorged sandflies were aspirated into incubation cages and maintained on fresh apple slices for up to 15 days in an insectary maintained at 26°C and 80% RH and photoperiod of 12 h:12 h [L: D].

On days 6, 10, and 15 postinfection, approximately 30% of the virus-exposed sandflies from each replicate were analyzed for the virus' presence. Individual sandflies were dissected in a cold chain (wet ice) to separate the legs and the salivary glands from the body containing the midgut. Individual bodies were placed separately in 1.5 ml microcentrifuge tubes containing 500 *μ*l of homogenizing media (HM), consisting of MEM, supplemented with 15% FBS, 2% L-Glutamine, and 2% antibiotic/antimycotic solution. Legs and salivary glands of individual sandflies were placed in separate 1.5 ml microcentrifuge tubes containing 300 *μ*l and 200 *μ*l of HM, respectively. The samples were immediately frozen at -80°C until assayed for virus recovery by cell culture.

### 2.5. Infection Success Assays

Individually dissected samples were homogenized using TissueLyserII (Qiagen, Inc., Valencia, CA) at 25 Hz for 3 min with an aid of sterile copper beads. The homogenates were clarified by centrifugation at 12,000 rpm for 10 min at 4°C. For oral susceptibility assessment, evidence of midgut infection was tested by inoculating 100 *μ*l of the supernatant of the clarified body samples onto 12-well plates of confluent Vero cells. The infected cells were incubated at 37°C for 1 h with frequent agitation for virus adsorption. The infected cells were maintained for up to 15 days on MEM as described earlier for virus amplification. For every positive body sample, their corresponding legs were screened for virus infection as evidence of dissemination, as described above for midgut infection in the body. Also, for every positive leg sample, the corresponding salivary glands were screened to ascertain virus transmission, as described above.

### 2.6. Bloodmeal Analysis

Engorged sandflies were morphologically identified by dissection and examination of the head and the posterior parts as proposed by Abonnenc and Minter [27]; details on this procedure are provided in Hassaballa et al. [25]. Individual abdomens of identified engorged sandflies were homogenized in 500 *μ*l phosphate buffer saline (PBS) with the aid of sterile copper beads using the TissueLyserII (Qiagen, Inc., Valencia, CA) at 25 Hz for 3 min. The homogenates were clarified by centrifugation, and the DNA was extracted using ISOLATE II Genomic DNA Kit (Bioline, Meridian Bioscience, Germany) according to the manufacturer's instructions.

A polymerase chain reaction of a 500 bp region of the 12S mitochondrial rRNA was performed in a 20 *μ*l reaction volume containing 4 *μ*l 1× Mytaq reaction buffer, 2 units of Mytaq DNA Polymerase (Meridian bioscience), 0.5 *μ*M concentration of both 12S-F GGGATTAGATACCCCACTATGC and 12S-R TGCTTACCATGTTACGACTT primers [28], and 2 *μ*l of genomic DNA template (~20 ng). The thermal cycling conditions used were as follows: initial denaturation was done at 95°C for 3 min, followed by 40 cycles of denaturation at 95^o^ for 20 s, annealing at 59°C for 30 s, and extension at 72°C for 30 s followed by a final extension at 72°C for 7 min. The PCR was carried out using a 96-well, 0.2 ml, 3-zoned VeriFlex Block SimplAmp Thermal Cycler (Thermo Fisher Scientific, MA). The amplicons were resolved on 1.5% agarose gel electrophoresis stained with ethidium bromide against a 100 bp DNA ladder (Bioline, Meridian Bioscience, Tennessee, USA). The PCR products of amplified samples were purified using the SureClean Plus kit (Bioline, Meridian Bioscience) and Sanger-sequenced using the forward primer only by a commercial firm (Macrogen Europe BV, Amsterdam, The Netherlands). Sequences obtained were edited using MEGA v.10 [29] and queried against the NCBI database using the BLASTn (http://www.ncbi.nlm.nih.gov/blast) search tool. Samples were assigned to bloodmeal host species when sequences had ≥98% identity [30, 31].

### 2.7. Ethical Approval

The study was approved by the Kenya Medical Research Institute Scientific Ethics Review Unit (KEMRI-SERU) (SERU protocol number 3312) and National Commission for Science, Technology and Innovation (NACOSTI) (permit number: NACOSTI/P/22/15808). NACOSTI (http://www.nacosti.go.ke) is mandated to accredit research institutions and approve all scientific research in Kenya. The current study complied with NACOSTI guidelines.

### 2.8. Data Analysis

The infection success was expressed as the proportion of infected sandflies' bodies among the orally exposed and tested sandflies. Dissemination was expressed as the proportion of the infected sandflies with infections in the legs of those with midgut infection and the transmission rates were expressed as the proportion of the sandflies with the virus in the saliva among the sandflies with disseminated infections. To reveal the infection progression across the incubation period, we performed Spearman's correlation analysis and generated a regression line for the midgut infection rates against the extrinsic incubation period. All the statistical analyses were in R version 4.1.0 [https://www.R-project.org/] at 95% level of significance.

## 3. Results

### 3.1. Sandfly Susceptibility to Oral Infection with NTPV

Forty-eight percent (48%, 255/528) of the orally exposed sandflies successfully obtained the viremic bloodmeal (Table 1). The titer of the virus in the bloodmeals reduced slightly within the 1 h of blood-feeding (Table 1).

**Figure 1:**
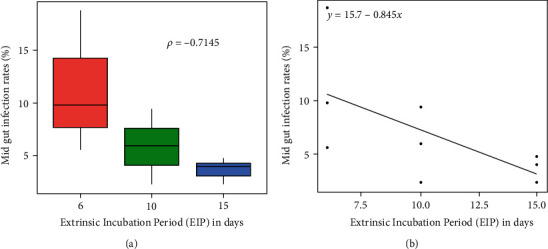
Midgut infection rates of NTPV in orally exposed *P. duboscqi* by incubation period. (a) Decreasing midgut infection rates with increasing incubation period. (b) Strong negative association between the midgut infection rates of NTPV in *P. duboscqi* and the extrinsic incubation period.

Of the 255 NTPV-exposed sandflies, 80% (205/255) survived to the 15 days postinfection and were all dissected or tested (Table 1). Spearman's correlation analysis showed a positive association between sandfly mortality rates and midgut infection rates (*ρ* = 0.61). Only midgut infections were observed in the exposed sandflies, and the rates were 10.7%, 5.4%, and 3.4% on 6-, 10-, and 15-days postinfection, respectively (Table 2). The overall midgut infection rate was 19.5% (40/205). There was no disseminated infection in the legs and consequently none in the salivary glands. Attempts to quantify the infection levels in individual positive midgut samples by plaque assay were unsuccessful. The infection rates were observed to decrease with increasing extrinsic incubation period (EIP) (Figure 1(a)). That is, infection rates were strongly negatively correlated with EIP (Spearman's correlation (*ρ* = −0.71) and regression line equation (*y* = 15.7 − 0.845x)) (Figure 1(b)).

### 3.2. Host Bloodmeal Sources

In total, 48 field-collected blood-fed sandflies were analyzed for bloodmeal sources represented by the species *S. schwetzi* (*n* = 22), *S. clydei* (*n* = 9), *S. squamipleuris* (*n* = 6), *S. antennata* (*n* = 6), *S. africana* (*n* = 2), and *Phlebotomus martini* (*n* = 3). Of these, data were successfully generated from 42 specimens (87.5%). The results show that humans and cattle are the most likely only bloodmeal host sources identified by PCR and sequencing. Most of the blood-fed sandflies had fed on humans (98%, 41/42) and only 2% on cattle by a sample of *S. clydei* (Table 3). Human bloodmeals were represented in all the sandfly species in proportion to the number analyzed (Table 3). There was no case of multiple hosts feeding in a single sandfly bloodmeal (Table 3).

## 4. Discussion

This study assessed the vector competence of the sandfly *P. duboscqi* to NTPV, a newly described virus of potential public health importance in Kenya. The findings of the study showed that the laboratory colonized *P. duboscqi* is an inefficient vector of NTPV when artificially challenged with the virus orally. The results also reveal a very high proportion of human feeding by diverse feral sandfly species from the focus where NTPV was originally isolated, highlighting a potential risk of human infection by pathogens that these sandflies might transmit.

We report a low level of oral susceptibility (midgut infection rate = 19.5%; 40/205) of *P. duboscqi* to NTPV. Low-level oral susceptibility of sandflies to viruses is an occurrence not uncommon in laboratory infection experiments [32]. However, the observed infection rate is lower than that observed for this species with the related *Phlebovirus* Rift Valley fever virus (RVFV) where about 50% of those orally exposed developed infections in the midgut [11, 12]. The low virus recovery from the midgut could have resulted from low infection levels of the midgut epithelial cells. Residual virus from the initial viremic bloodmeal could also be associated with the low-level recovery of the virus from the midgut [10]. Residual virus unable to infect the midgut cells may lose viability over time, which could explain the strong negative association between infection rates and EIP in this study. In vector competence of the sand fly *L. longipalpis* to RVFV, both the virus titers, and the number of infected sandflies reduced with increasing EIP [10].

*Phlebotomus duboscqi* might have a strong midgut escape barrier to NTPV likely limiting the possibility of disseminated infection across the midgut. In fact, NTPV failed to disseminate after midgut infections in the fly. The result is consistent with previous findings that reported nondisseminated midgut infections of RVFV in *L. longipalpis* at comparable or higher ingested titers (10^4^-10^7.2^ pfu/ml) even though 60% of the sandflies were susceptible [10]. *Phlebotomus duboscqi* likely possesses a strong midgut escape barrier to NTPV potentially conferred by the genetics of the vector [11]. Alternatively, dissemination may have been impeded by the formation of a peritrophic membrane that forms about 48 h after the bloodmeal, enveloping the bloodmeal and blocking the progression of the virus to secondary tissues as has been reported in experimental infection of *P. pernicious* with Toscana virus [33]. Very low dissemination rates were also observed in *P. duboscqi* orally infected with RVFV with only 6 of the 145 exposed flies transmitting the infection [12] despite a 35% midgut infection outlining the existence of a strong midgut escape barrier in *P. duboscqi.*

For a competent vector, the ingested virus infects and replicates in the midgut to higher titers and disseminates progressively to the salivary glands of the vector within the EIP. Sandfly-borne phleboviruses have an EIP of about 7 days after which the sandflies remain infective throughout their lifespan [32]. *P. duboscqi* had no virus infection in the salivary glands reminiscent of the case of RVFV in this same sandfly species with a comparable ingested virus titer [11]. *P. duboscqi* could equally possess a strong salivary gland infection barrier to NTPV, although this remains to be verified. The dissemination of a virus from the midgut after overcoming the midgut escape barrier is suggested to be enough to guarantee vector competence of *P. duboscqi* to viruses [12]. In support of this hypothesis, nearly all disseminated infections from virus ingestion or infections introduced by intrathoracic inoculation were found to surmount the midgut infection and escape barriers [34] and always transmissible in laboratory studies [11, 12]. Noteworthy, *in vitro* vector competence experiments may poorly represent the natural transmission of a virus. The NTPV dose ingested from a host during natural feeding is not unknown. However, the titers used in this study closely resemble those of RVFV used to assess *P. duboscqi* vector competence [12]. To assess transmission by bites, previous studies on mosquitoes use forced salivation by inserting the proboscis of immobilized mosquitoes into capillary tubes containing either blood or homogenization media [35–37]. This approach has not been demonstrated to be successful in sandflies. To date, there is no data on suitable susceptible animal models for NTPV to allow for experimental validation of transmission success. Dissection and testing of the salivary glands were adopted to recover the virus for evidence of transmission.

In this study, a high human feeding rate was observed among several sandfly species collected from Baringo, where NTPV was initially detected. The result is consistent with previous reports describing >90% human feeding rates by feral sandflies in the same ecology [5, 6]. However, these earlier studies did not associate bloodmeals with individual sandfly species. *S. schwetzi* was predominant among the blood-fed specimens likely reflecting its abundance in the sandfly fauna of this ecology [25]. Despite the association of *Sergentomyia* species with human feeding [5, 6], they have not been implicated in the transmission of human pathogens [38]. NTPV was likely isolated from *Sergentomyia* species (e.g. *S. schwetzi*) based on DNA barcoding of sandflies in the infected pool [5, 6]. The overwhelming association with human biting activities as demonstrated in this study requires a reevaluation of their role in pathogen spread including NTPV. The few *P. martini* analyzed all had human-derived bloodmeals agreeing with previous literature regarding its feeding habit [14, 39]. No *P. duboscqi* was represented among captured blood-fed samples perhaps attributed to low fly occurrence at the time of sampling [25]. Since the transmission of sandfly-associated phleboviruses has always been almost obligately associated with the *Phlebotomus* species [8], further studies should also investigate the role of *P. martini* in the transmission of the novel phleboviruses including use of recently colonized populations of *P. duboscqi*. Sandflies exhibiting high feeding on humans have been incriminated as vectors of sandfly-borne pathogens (e.g., phleboviruses) affecting humans in the same habitat [18, 40]. The high human blood-feeding of *Sergentomyia* species and their detection in positive pools of novel phleboviruses detected in this focus [5, 6] highlight the need to determine their roles in the transmission of these viruses.

## 5. Conclusion

This study demonstrated that *P. duboscqi* is an inefficient vector of NTPV, and that several *Sergentomyia* species show a strong feeding preference to humans as blood-meal sources in Baringo County, Kenya. We recommend the establishment of different sandfly species colonies to allow further studies to examine the role of *Sergentomyia* species and *P. martini* in the transmission of NTPV and other novel phleboviruses detected in this study and other arbovirus foci of Kenya.

## Figures and Tables

**Table 1:** Infection of laboratory-reared *P. duboscqi* with NTPV by membrane feeding.

Experiment	Percent blood-fed (no. fed/no. exposed)	Percent tested (no. survived/no. fed)	Prefeeding titer	Postfeeding titer
Replicate 1	45.5 (97/213)	92.8 (90/97)	5.72	5.15
Replicate 2	43.5 (87/200)	73.6 (64/87)	4.6	4.3
Replicate 3	61.7 (71/115)	71.8 (51/71)	6.6	6.3
Total	48.3 (255/528)	80.4 (205/255)		

Prefeeding titer: titer of the viremic bloodmeal before exposure to the sandflies; postfeeding titer: titer of the viremic bloodmeal after 1 h exposure to the sandflies. Titers are expressed as Log10 pfu/ml values; pfu/ml: plaque-forming units per 1 ml of blood.

**Table 2:** Infection, dissemination, and transmission rates of NTPV in *P. duboscqi* orally infected with ≈10^6.0^ pfu/ml NTPV.

Vector competence index	6DPI	10DPI	15DPI
Infection rate	10.7 (22/205)	5.4 (11/205)	3.4 (7/205)
Dissemination rates	0	0	0
Transmission rate	0	0	0

DPI: days postinfection; n: number of positive samples; infection rate: the percentage of the tested sandflies with the virus recovered in the midgut; dissemination rate: the percentage of the tested sandflies with the virus recovered from the legs; transmission rate: the percentage of the sandflies with the virus recovered from the salivary gland.

**Table 3:** Bloodmeal sources of phlebotomine sandflies from Rabai, Baringo County, Kenya.

Sandfly species	No. analyzed (no. successful/no. processed)	Proportion feeding on human	Proportion feeding on cattle
*P. martini*	3/3	3/3	0
*S. Africana*	1/2	1/1	0
*S. antennata*	5/6	5/5	0
*S. clydei*	8/9	7/8	1/8
*S. schwetzi*	21/22	21/21	0
*S. squamipleuris*	4/6	4/4	0
Total	42/48	41/42	1/42

## Data Availability

All data supporting the conclusions of this article are included within the article.
